# Toward a Typology of Counterproductive Employees: A Person-Oriented Investigation of Counterproductive Work Behavior

**DOI:** 10.17505/jpor.2023.25256

**Published:** 2023-06-17

**Authors:** Justin Travis, S. Bartholomew Craig

**Affiliations:** 1 University of South Carolina – Upstate, USA; 2 North Carolina State University, USA

**Keywords:** person-oriented approach, counterproductive work behavior, dark triad, latent profile analysis

## Abstract

The study of counterproductive work behavior (CWB), intentional actions by employees that are deleterious to the organization and/or its stakeholders, has produced research on the dimensionality of CWB, as well as its situational and dispositional antecedents. Absent from these advancements have been investigations into the potential utility of a taxonomy of counterproductive employee types—a “person-oriented” approach. Our latent profile analysis (*N* = 522) suggested a four-profile solution which included one profile with uniformly low rates across CWBs (here termed “Angels;” 14% of the sample), and three profiles with higher CWB rates but which were distinguishable by different CWBs being most frequent in each group. Specifically, one profile was distinguished from the Angels group by higher rates of less severe CWBs (misuse of time/resources and poor attendance; 33% of the sample). The other two of the three counterproductive profiles were similar to each other except that one was characterized by higher drug use than the other (14% of the sample). The profiles also differed significantly on narcissism, psychopathy, and Machiavellianism, and on self-reports of prior arrest and censure by employers. Provided these distinctions among profiles, the treatment and assumptions of employee counterproductivity in research and practice should be revisited, particularly when using models assuming a homogenous, monotonic relationship between counterproductive behaviors across employees. Implications for our conceptual understanding of counterproductivity and applied interventions aimed at reducing CWBs are discussed, alongside recommendations for future person-oriented research on CWB.

## Introduction

Counterproductive work behavior (CWB) refers to discretionary acts by organization members that harm or intend to harm their organization, coworkers, or other stakeholders (Spector et al., 2006). Several streams of research have documented, classified and predicted CWBs, prompted in large part by the repercussions of counterproductivity in the workplace. Most, if not all, of these research streams have adopted a variable-centered approach that reports correlates of CWB, and taxonomies based on perceived similarities among specific behaviors. While such research has extended scientific understanding of CWBs, it has not addressed the possibility that there are configurations of counterproductive tendencies that manifest themselves within-persons. Currently, there is little published research investigating whether there are different patterns of counterproductive behavior among employees. This limitation may limit both research and interventions by neglecting how CWBs do, or do not, combine within-persons to produce distinct types of counterproductive employees. Scientists have long addressed the frequencies and amounts of undesirable behavior, whether specific bad behaviors (e.g., theft) or behavior in aggregate (e.g., CWB), and related those findings to other variables. To extend this research, we propose that an important next step is to understand how and which behaviors may operate in conjunction with each other, and establish empirical relationships among those various CWB blends with individual differences in personality.

The totality of CWB research to date has taken a variable-centered approach, thereby enhancing understanding of the structure, antecedents, and consequences of CWB by focusing on relations among variables. Nevertheless, there is an increasingly vocal call to complement traditional variable-centered research with person-oriented approaches (Asendorpf, 2006; Magnusson, 2003; Wang & Hanges, 2011). Among other advantages, a person-oriented approach provides an opportunity to identify distinct types of counterproductive employees, based on their within-person patterns across CWB dimensions. Despite considerable research into CWB antecedents and various distinctions among those, there has been little attention given to how CWBs may co-occur within persons. Such an approach offers a nuanced perspective on how various counterproductive behaviors combine into distinct configurations, much like person-oriented approaches to personality profiles (Asendorpf, 2006; Shoss & Witt, 2013).

Because of their reliance on tests of statistical significance, variable-oriented approaches will only identify within-person co-occurrences that hold in general across the entire sample being studied, whereas a person-oriented approach can identify co-occurrences that hold for different subsets of the sample. Further, such examination of separate subpopulations amounts to an examination of higher-order interactions that would require prohibitively large sample sizes with a variable-oriented approach (e.g., five-way or higher interactions). If there exist subpopulations among counterproductive employees that manifest varying patterns of co-occurrence, then describing and linking these with dispositional antecedents can have implications for selection, assessment, and interventions aimed at reducing CWB. Indeed, recent research has taken profile approaches to classifying the dispositional antecedents of CWBs (e.g., dark triad profiles; Nguyen et al., 2021), yet we found no research that applied the same approach to counterproductive work behaviors themselves. Considering the aforementioned limitations of variable-centered approaches, we believe it prudent to contribute to the work in this area by investigating potential patterns of co-occurrence that differ between employees. The present study provided a person-oriented analysis of CWB that complements extant variable-centered research by examining whether individuals display different patterns of CWB and linking those *patterns*—rather than individual variables—to commonly studied dispositional antecedents.

### Co-occurrence of CWBs

Previous studies have examined behavior co-occurrence that was uniform across samples (e.g., Bennett & Robinson, 2000) or perceived similarity/likelihood of co-occurrence (e.g., Gruys & Sackett, 2003; Robinson & Bennet, 1995). These studies have produced instruments and taxonomies using the variable-centered approach that have advanced the study of counterproductivity. For example, Bennett and Robinson (2000) identified the key dimensions of CWB as those behaviors aimed at the organization (organizational deviance or CWB-O), and those aimed at other individuals at work (interpersonal deviance or CWB-I). Two other influential papers in the CWB literature instead specify the behavioral domain, or type, as important dimensions of CWB (Gruys & Sackett, 2003; Spector et al., 2006). In particular, Gruys and Sackett (2003) offered what is perhaps the most fine-grained classification of CWB content dimensions from the results of two studies, based on perceived co-occurrence, resulting in 11 categories of CWB.

It is important to note that while previous researchers have looked at co-occurrence across an entire sample (Bennett & Robinson, 2000) or perceived similarity/co-occurrence (Gruys & Sackett, 2003), we found no evidence of previous studies exploring co-occurrence in or between subpopulations within a sample. A consequence of the traditional variable-oriented approach, and its associated reliance on significance testing, is that relations among variables will only be detected as significant if they hold in general across the entire sample. If a sample is composed of multiple subgroups, and a given relation holds in some subgroups but not others, then such a relation will not be found to be significant at the level of the entire sample.

### A person-oriented approach to CWB

The traditional approach to studying organizational phenomena, and psychological phenomena more broadly, has been variable-centered (Wang & Hanges, 2011). The variable-centered approach seeks to identify relations among variables, where an individual’s location on some latent variable (e.g., narcissism) is considered in comparison with others’ locations, typically in terms of rank-order, and is then related to that individual’s location on some other latent variable (e.g., CWB) also considered in comparison with others’ locations (Magnusson, 2003). The focus of the variable-centered approach is variables and their relationships with each other at the group level. Importantly, relationships between variables that emerge at the group level may not be necessarily descriptive of relationships between variables at the individual level within the sample or population (the ecological fallacy; Robinson, 1950). By identifying relations among variables, variable-oriented approaches can inform hypotheses regarding outcomes for individuals with specific within-person configurations of variables, but they provide no explicit tests of those hypotheses, nor do they identify which configurations actually exist in nature.

A notable limitation of a variable-centered approach is the absence of attention to individuals’ standing on multiple factors at once. For example, CWBs are most frequently measured using instruments that tap into multiple dimensions, and scores are either reported at the dimension level (e.g., inter-personal deviance or theft) or aggregated across dimensions and reported at the superordinate level (e.g., total CWB). Additionally, research testing the dispositional basis of CWB has related specific traits to those dimensions or to total CWB. However, the tests of such relations for statistical significance inherently assume a homogenous population wherein those relations function in a similar fashion across the sample (or population) as a whole. Without evidence of multivariate homogeneity in the population of interest, these assumptions may not hold and subsequent inferences may be problematic. If there are latent subpopulations with regard to patterns of counterproductivity, then there may be different outcomes for different profiles of CWB that are obscured without explicit person-oriented analyses.

In brief, a person-oriented approach seeks to understand intraindividual content and processes as they occur at the organismic or individual level (Cervone, 2005). While variable-centered approaches have certainly advanced psychological understanding—evidenced by the variable-centered literature that underpins the current literature re-view—these approaches may overlook important configurations of behaviors within individuals. Thus, exploring possible configurations of counterproductive tendencies within-individual can complement the existing CWB literature.

Person-oriented approaches, sometimes called profile, person-centered, pattern-based, or configural, are not new (i.e., Cronbach & Gleser, 1953); however, they have recently garnered increased attention from scholars in the personality (Asendorpf, 2006; Shoss & Witt, 2013), school (Davison & Kuang, 2000), developmental (Magnusson, 2003), and industrial/organizational psychology literatures (Morin et al., 2011). For the purposes of this paper, person-oriented, person-centered, configural, and profile approach will be used interchangeably as the language that follows will align closely with that used in empirical research (e.g., elevation/level, shape, and scatter; Cronbach & Gleser, 1953), although we acknowledge that our approach is only one basic form of the many person-oriented methods available to researchers (see Bergman & Lundh, 2015).

### The Dark Triad

Although broad and narrow personality traits have been used to predict counterproductive work behavior, scientists have recently turned to personality traits that are conceptually related to deviance and counterproductivity. Specifically, researchers have begun studying the predictive validity of the so-called “dark triad” traits: narcissism, Machiavellianism, and psychopathy (Paulhus & Williams, 2002). Given that CWB is typically defined by its deviance from organizational interests and/or social norms, it seems reasonable that individual differences that are conceptually related to deviance and unethical behavior would predict counterproductive behavior at work. “Dark” personality traits in general have sparked interest from personnel and organizational psychologists (e.g., Wu & LeBreton, 2011); however, it is the dark triad that has received the most attention.

The dark triad refers to subclinical ranges of three personality traits: narcissism, Machiavellianism, and psychopathy (Paulhus & Williams, 2002). The dark triad dimensions, like the traits of the FFM, are only partially independent of each other. Because each of these three dark traits is a composite of narrow facets from diverse larger traits, their overlap with each other is both expected and cumbersome. For example, some research has found narcissism, Mmachiavellianism, and psychopathy to be negatively correlated with agreeableness (Paulhus & Williams, 2002), while other studies have found only psychopathy and Mmachiavellianism to be negatively correlated with agreeableness (Lee & Ashton, 2005). The interrelatedness of the three traits has also been supported by at least two recent meta-analyses that report a range of intercorrelations between .23 and .58, with both re-porting the Machiavellianism-psychopathy relationship to be the strongest (Muris et al., 2017; O’Boyle et al., 2012). Collectively, the small-to-medium relationships within the dark triad, and their relationships with external criteria, prompt the following discussion to describe each trait separately while acknowledging their overlap.

#### Narcissism

While narcissism at the clinical level refers to, “a pervasive pattern of grandiosity (in fantasy or behavior), need for admiration, and lack of empathy, beginning by early adulthood and present in a variety of contexts” (American Psychiatric Association, 2013), research investigating narcis-sism-CWB relationships most frequently focuses on assumed subclinical levels of narcissism. Some of the critical features underlying narcissism are grandiosity and a simultaneous insensitivity to others’ concern and hypersensitivity to perceived criticism of their exaggerated self.

Such characteristics lend themselves to a dismissive attitude toward workplace rules and norms, leading to increased rule-breaking behavior (Penney & Spector, 2002). Indeed, narcissism has been found to be the strongest predictor of CWB beyond the FFM and the other dark triad traits (Grijalva & Newman, 2015; O’Boyle et al., 2012).

#### Machiavellianism

Whereas narcissism entails a self-enhanced and entitled view of oneself, Machiavellianism captures a self-interested and manipulative approach to social interaction. Individuals high in Machiavellianism behave callously toward others, are suspicious of others' motives, and their actions frequently conflict with the interests of others (Christie & Geis, 1970; Wilson et al., 1996). The cynical and manipulative nature of Machiavellianism is thought to lend itself to increased levels of broadly unethical behavior, such as lying and stealing (Jones & Paulhus, 2009).

#### Psychopathy

Individuals high in psychopathy are impulsive and deceptive, as well as resistant to the anxiety, shame and guilt that are typically produced by deviant behavior. Although there remains debate regarding the factor structure of psychopathy, Cooke and Michie (2001) proposed three primary dimensions of psychopathy: arrogant and deceitful interpersonal style, deficient affective experience, and impulsive and irresponsible behavioral style. The characteristics of psychopathy lend themselves to impulsive, callous behaviors that would theoretically be expected to harm others. Indeed, a recent meta-analysis reported relationships between psychopathy and a diverse pool of undesirable factors, such as aggression and delinquency (*r* = .39) and antisocial tactics (e.g., lying or cheating, *r* = .32; Muris et al., 2017).

In summary, the dark triad represents three related but distinct personality factors that (a) are amalgamations of narrow personality traits that capture variance from several FFM factors (Lee & Ashton, 2005; Wu & LeBreton, 2011); (b) are conceptually proximal to varieties of deviant outcomes (Paulhus & Williams, 2002); and (c) predict counterproductive work behavior (O’Boyle et al., 2012). Given the aforementioned research on the dark triad and CWB, the current study includes the dark triad for the purpose of integrating possible CWB profiles with the emergent literature on dark personality at work.

### The Present Study

The purpose of this study was to investigate the within-person co-occurrence of CWB using a person-oriented methodological approach. Examining CWB “types” defined by the pattern of frequencies across different forms of CWB provides a novel contribution to our knowledge in this area. Building on extant research and answering the call for person-oriented approaches to CWB, the present study aimed to identify distinct profiles of CWB using a broad set of behaviors from common CWB measures. Specifically, this study tested the assumption that diverse counterproductive behaviors are manifested in a uniform fashion across individuals by investigating the possibility of subgroups where these behaviors combine in unique ways.

To the authors’ knowledge, no previous study has investigated whether subgroups of counterproductive employees exist, in terms of the frequencies (levels) and types (shapes) of counterproductive behaviors they exhibit.

*Research Question 1*: How many distinct profiles of counterproductive work behavior exist among working adults?

*Research Question 2*: What are the characteristics (level and shape) of each profile and what proportion of the sample is classified in each of these profiles?

Considering the recent support for a reflective model of a general CWB construct (Marcus et al., 2016), a pattern-based approach may provide further clarity on how dispositional antecedents relate to the configuration of CWB within-person. Theories that propose internal/dispositional causes of counterproductivity may benefit from empirical evidence linking those dispositions to profiles of CWB. Thus, the current study also tested whether common trait correlates of CWB are predictive of profile membership.

*Research Question 3:* How do individual differences in dark triad traits relate to profile membership?

Because of the clandestine nature of many CWBs, objective indicators of CWB frequency are notoriously difficult to implement in research, and researchers have traditionally been forced to rely on participants’ self-reports of misdeeds as a result. To augment self-reports of CWB, the current study also collected self-reports of received sanctions that would theoretically be objectively verifiable. Although the self-reported sanctions were not actually verified in the current study, we expected that their recall might activate different memory systems and self-presentation motives, such that their inclusion would allow further validation of any typology we derived.

*Research Question 4*: Does profile membership predict previous organizational sanctioning or disciplinary action?

*Research Question 5:* Does profile membership predict non-work deviance (e.g., arrest history)?

## Method

### Participants and Procedure

Participants were recruited from Amazon’s Mechanical Turk (mTurk) platform. Participants were required to be (a) 18 years or older, (b) based in the United States and fluent in English, and (c) currently employed full-time at 30 or more hours per week. Studies comparing data collected from mTurk samples to traditional student and employee samples have largely supported its use in psychological research (Behrend et al., 2011; Buhrmester et al., 2011; Casler et al., 2013). After providing informed consent, participants received a battery of questionnaires including measures of the dark triad, CWB, previous work discipline and arrest history, and demographic information.

Prior to testing research questions, data were screened for careless responding (Meade & Craig, 2012). Specifically, careless participants were identified by three different indicators: (a) an instructed response item (“Please select ‘disagree’”); (b) maximum identical responses across 15 or more sequential items (“LongString”); and (c) inspection of openended, forced-response text. Of the 640 participants providing data, 36 participants failed the instructed response and 11 were identified as careless responders via maximum LongString. The remaining 593 cases were further screened for careless responding by inspecting the open-ended text responses provided at the end of the survey in response to, “Take a moment to think back on the negative work behaviors that were described above. Please explain why you did, or did not, choose to do those behaviors.” A conservative screen was performed whereby nonsensical, blank, or uninterpretable responses were identified (*n* = 71) and removed from the data set.

The final sample (*N* = 522) was 56% female, and the average age and tenure in the current job were 37.3 years (*SD* = 10.8) and 80.5 months (*SD* = 73.5), respectively. The most commonly reported employment industries were healthcare or social assistance (12%), finance or insurance (11%), and educational services (11%). An administrative error left racial demographic data uncollected.

### Measures

#### Dark Triad

The 27-item Short Dark Triad scale (SD3; Jones & Paulhus, 2014) was used to measure Machiavellianism (9 items; α = .85), psychopathy (9 items; *α* = .81), and narcissism (9 items; *α* = .75). Participants were asked to consider each item and respond using a 5-point Likert scale anchored from 1 (*disagree strongly*) to 5 (*agree strongly*).

#### Counterproductive Work Behavior

Sixty-five items from Gruys and Sackett’s (2003) scale were used to measure 11 dimensions of CWB: theft and related behavior (TRB, 10 items; α = .96), destruction of property (DP, 4 items; α = .94), misuse of information (MI, 5 items; α = .90), misuse of time and resources (MTR, 13 items; α = .89), unsafe behavior (UB, 4 items; α = .93), poor attendance (PA, 5 items; α = .92), poor quality work (PQW, 3 items; α = .89), alcohol use (AU, 3 items; α = .92), drug use (DU, 4 items; α = .93), inappropriate verbal actions (IVA, 8 items; α = .94), and inappropriate physical actions (IPA, 6 items; α = .94). While the original IPA scale had seven items, an error in the survey entry left the final item, “Make unwanted sexual advances toward a customer” off the survey that the participants completed. In their scale development paper, Gruys and Sackett (2003) asked participants to rate whether they would engage in each of the behaviors using a 7-point scale. For the purposes of the current study, and similar to other research (e.g., Bragg & Bowling, 2018), participants were instructed to report the frequency with which they actually engage in the behaviors using an 8-point scale anchored from 1 (*never*) to 7 (*daily*), with an 8th option “Not Relevant,” which was treated as missing data during analyses.

Although this measure has been used in research somewhat less often than its counterparts (e.g., Bennett & Robinson 2000; Spector et al., 2006), it has been considered one of the most inclusive measures of CWB by some organizational scholars (Marcus, et al., 2016; Wu & LeBreton, 2011).

#### Previous Disciplinary Action

In order to assess previous disciplinary action, participants were asked two questions developed for the current study: “How many times have you been disciplined or punished for breaking rules at any job?” and “How many times have you been fired or terminated from any job?” Response options included “zero” “one” “two” “three” and “four times or more.” Although these items represent relatively discrete phenomena, they are not necessarily independent and were positively correlated (*r* = .55, p < .01).

#### Previous Arrest History

Participants were asked to respond to the question, “How many times have you been arrested for any reason other than traffic offenses?” Response options included “zero”, “one” “two”, “three,” and “four times or more.”

## Results

### Measurement Model

Descriptive statistics and intercorrelations for all study variables are shown in Table 1. To evaluate the functioning of the study’s measures, a confirmatory factor analysis (CFA) was conducted using the “lavaan” package in R (Rosseel, 2019). Due to the high ratio of items to constructs, items were parceled within scales using the random method (see Little et al., 2013 for a discussion). Random numbers were generated and used to group items within construct, a method endorsed by scholars when there is little theoretical justification for other methods of parceling (Little et al., 2002). This random parceling method was followed for all scales with more than five items. Scales with five or fewer items were estimated as typical in CFA whereby the item-level indicators were modeled on their respective latent variables.

**Table 1:** Descriptive Statistics and Intercorrelations for Study Variables

	*M*	*SD*	1	2	3	4	5	6	7	8	9	10	11	12	13	14	15	16	17	18	19	20
1 Age	37.3	10.76	1																			
2 Sex	-	-	.09	1																		
3 Tenure	80.5	73.50	.52	.04	1																	
4 Mach	2.84	.78	-.20	-.12	-.06	(.85)																
5 Narc	2.66	.65	-.19	-.16	-.17	.40	(.75)															
6 Psyc	2.15	.73	-.31	-.34	-.15	.62	.45	(.81)														
7 TRB	1.43	1.03	-.23	-.16	-.13	.25	.27	.50	(.96)													
8 DP	1.37	1.07	-.22	-.16	-.12	.21	.25	.46	.93	(.94)												
9 MI	1.50	1.07	-.24	-.14	-.14	.27	.26	.51	.94	.91	(.90)											
10 MTR	2.05	1.12	-.21	-.12	-.09	.31	.20	.45	.81	.76	.81	(.89)										
11 UB	1.46	1.12	-.23	-.16	-.14	.23	.26	.47	.93	.91	.90	.78	(.93)									
12 PA	1.65	1.11	-.21	-.14	-.14	.27	.24	.49	.89	.85	.89	.83	.87	(.92)								
13 PQW	1.52	1.16	-.24	-.18	-.13	.24	.24	.51	.92	.89	.91	.80	.88	.86	(.89)							
14 AU	1.47	1.15	-.24	-.19	-.14	.26	.26	.51	.89	.88	.88	.75	.85	.85	.86	(.92)						
15 DU	1.44	1.16	-.23	-.18	-.13	.25	.23	.50	.90	.89	.88	.76	.87	.86	.87	.90	(.93)					
16 IVA	1.50	1.07	-.22	-.20	-.11	.26	.26	.53	.91	.91	.90	.81	.88	.84	.90	.86	.90	(.94)				
17 IPA	1.38	1.09	-.23	-.17	-.11	.22	.26	.49	.93	.93	.91	.76	.90	.84	.91	.88	.91	.91	(.94)			
18 Disc	.47	.90	-.07	-.19	-.03	.18	.10	.34	.42	.37	.41	.43	.38	.45	.39	.39	.37	.44	.35	1		
19 Fired	.47	.81	.04	-.14	-.06	.21	.09	.32	.41	.38	.38	.36	.40	.40	.38	.39	.42	.41	.36	.55	1	
20 Arrest	.30	.75	-.08	-.09	-.05	.24	.12	.40	.47	.43	.42	.43	.44	.46	.50	.49	.48	.49	.47	.45	0.46	1

*Note*. Values along the diagonal are coefficient alphas for multi-item scales. Mach = Machiavellianism, Narc = narcissism, Psych = psychopathy, TRB = theft and related behaviors, DP = destruction of property, MI = misuse of information, MTR = misuse of time and resources, UB = unsafe behavior, PA = poor attendance, PQW = poor quality work, AU = alcohol use, DU = drug use, IVA = inappropriate verbal behaviors, IPA = inappropriate physical actions, Disc = previous disciplinary experiences, Fired = previous number of terminations, and Arrest = previous arrest history.

Using the maximum likelihood estimation procedure and allowing the latent variables to covary, results suggested the data demonstrated marginal fit (*χ^2^*(1036) = 4250.04, *p* < .001, CFI = .881, RMSEA = .083 (.08-.086), SRMR = .046; Byrne, 2011; Hu & Bentler, 1998; 1999) with the hypothesized 14-factor model (three dark triad factors and eleven CWB factors). All factor loadings were above .40 and statistically significant (*p* < .001), and no modifications were performed to the model.

### Profile Analyses

Research question 1 asked: How many distinct profiles of counterproductive work behavior exist among working adults? Research question 2 asked: What are the characteristics (level and shape) of each profile and what proportion of the sample is classified in each profile? To investigate both research questions, latent profile analysis (LPA) was conducted using R via the “mclust” package (Scrucca et al., 2016). LPA is a model-based analytic method, from a larger class of mixture models that can be used to identify and describe unobserved subpopulations (Wang & Hanges, 2011). Using maximum likelihood estimation, LPA produces probabilities of latent class membership (unobserved subpopulations) with continuous data, and each model is estimated with additional latent profiles added iteratively. Model fit indices (Bayesian Information Criterion [BIC], Integrated Complete-data Likelihood criterion [ICL], and the Boot-strapped Likelihood Ratio Test [BLRT]) were compared between fourteen models that varied in parameterization constraints (e.g., varying vs. equal volume, varying vs. equal shape; see Scrucca et al., 2016). Research has found the BIC to be a robust indicator of the number of profiles at varying sample sizes (Nylund et al., 2007). Considering the lack of previous empirical and theoretical work concerning the current research questions, identifying the optimal solution, or number of profiles, was guided by the preponderance of evidence from multiple model fit indices.

**Figure 1:**
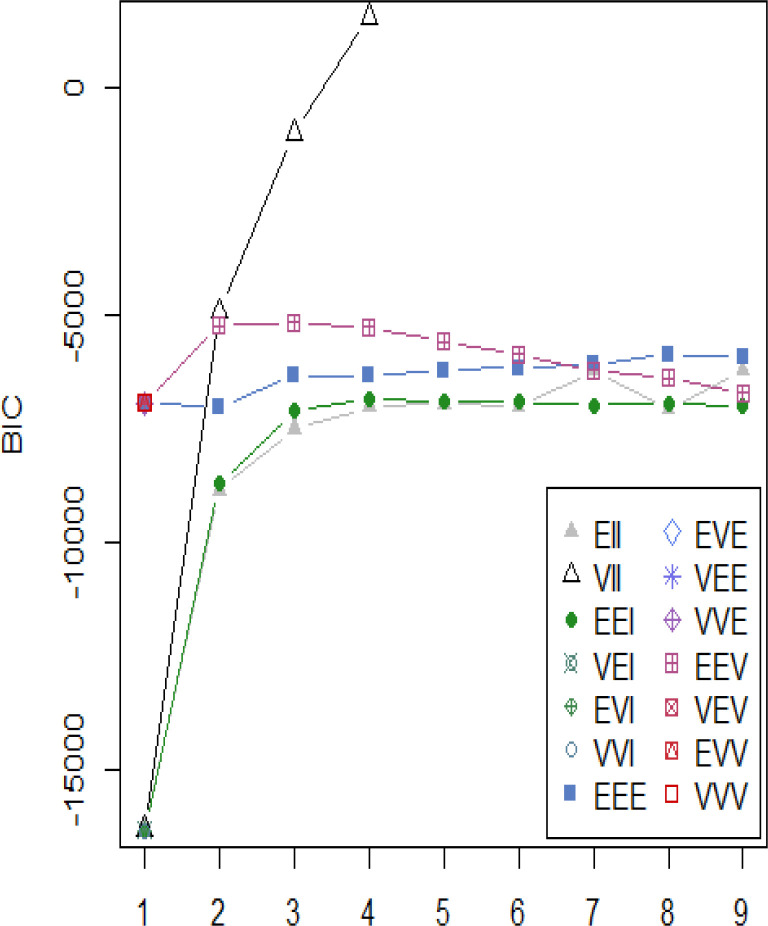
Bayesian Information Criterion (BIC) values for models with 1-9 profiles. “VII” refers to spherical, equal shapes with varying volume (Scrucca, Fop, Murphy, & Raftery, 2016).

The results of the latent profile analysis suggested that a four-profile solution was superior to alternative models. Specifically, a four-profile solution with spherical, equal shapes and varying volume (“VII” model in mclust; Scrucca et al., 2016) was identified as the optimal model by the two descriptive fit criteria (BIC = 1563.35, ICL = 1551.17), as well as the BLRT comparison of a four-profile solution to a three-profile solution (BLRT = 2647.19, *p* < .001). As there was no previous literature from which to predict or organize potential CWB profiles, and the three criteria converged on the four-profile solution as providing optimal fit with the data, the four profiles were used as the basis of subsequent analyses involving profile membership. A plot of each model’s four-profile BIC values is shown in Figure 1.

**Figure 2:**
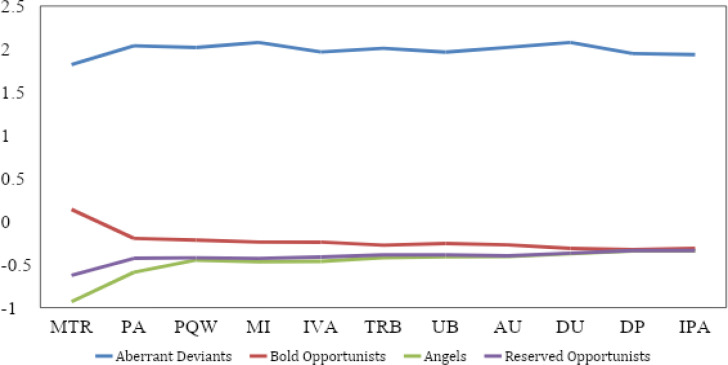
Profile z-scores for each dimension of counterproductive work behavior. TRB = theft and related behaviors, DP = destruction of property, MI = misuse of information, MTR = misuse of time and resources, UB = unsafe behavior, PA = poor attendance, PQW = poor quality work, AU = alcohol use, DU = drug use, IVA = inappropriate verbal behaviors, IPA = inappropriate physical actions.

Research question 2 was addressed by calculating the means and standard deviations for each profile across all CWB dimensions, as well as the proportion of the sample classified into each profile. Table 2 and Figure 2 display this information for the four profiles. As Table 2 reports, there were differences and similarities across profiles, with peaks (e.g., misuse of time and resources, poor attendance) and valleys (destruction of property, unsafe behavior, inappropriate physical actions) shared among the three profiles that reported counterproductive behaviors.

**Table 2:** Profile Distributions, Means, and Standard Deviations

Profile number (*n*)				
Dimension	1 (*69*)	2 (*191*)	3 (*67*)	4 (*162*)
TRB	3.50(1.46)	1.15(.23)	1.00(0)	1.03(.08)
DP	3.46(1.67)	1.01(.07)	1.00(0)	1.00(.03)
MI	3.72(1.39)	1.24(.33)	1.00(0)	1.04(.10)
MTR	4.09(1.10)	2.21(.54)	1.01(.03)	1.36(.26)
UB	3.66(1.67)	1.17(.33)	1.00(0)	1.02(.07)
PA	3.91(1.31)	1.43(.43)	1.00(0)	1.18(.20)
PQW	3.85(1.55)	1.27(.48)	1.00(0)	1.03(.10)
AU	3.78(1.58)	1.16(.34)	1.00(0)	1.01(.06)
DU	3.86(1.61)	1.07(.26)	1.00(0)	1.01(.05)
IVA	3.61(1.41)	1.24(.37)	1.00(0)	1.05(.11)
IPA	3.50(1.74)	1.03(.14)	1.00(0)	1.01(.04)

*Note*. TRB = theft and related behaviors, DP = destruction of property, MI = misuse of information, MTR = misuse of time and resources, UB = unsafe behavior, PA = poor attendance, PQW = poor quality work, AU = alcohol use, DU = drug use, IVA = inappropriate verbal behaviors, IPA = inappropriate physical actions.

The third profile had the fewest members at 14% of the sample (*n* = 67) and was marked by near uniform (non)reporting of CWBs (i.e., “never” endorsed across CWB scales). Accordingly, this profile was labeled “angels” and most interpretation of the results that follow focused on comparing the other three profiles that did report some levels of CWBs.

The first profile was marked by comparatively high levels of CWB across measures and accounted for 14% (*n*= 69) of the sample that was classified. These individuals engaged in much higher amounts of CWB than individuals with other profiles; however, some of the shape of their CWBs was similar with the other two profiles showing CWBs. Whereas profiles 2 and 4 reported almost no drug use, destruction of property, or inappropriate physical actions, drug use was one of the highest reported CWBs for profile 1. In fact, drug use and misuse of information were both magnitudes higher than the sample’s total mean (*z*= 2.08 and *z*= 2.08, respectively). Given the high levels of CWBs across this profile, its relative departure in shape from other profiles, and its separation from the other three profiles, this profile was labeled “aberrant deviants.”

**Figure 3:**
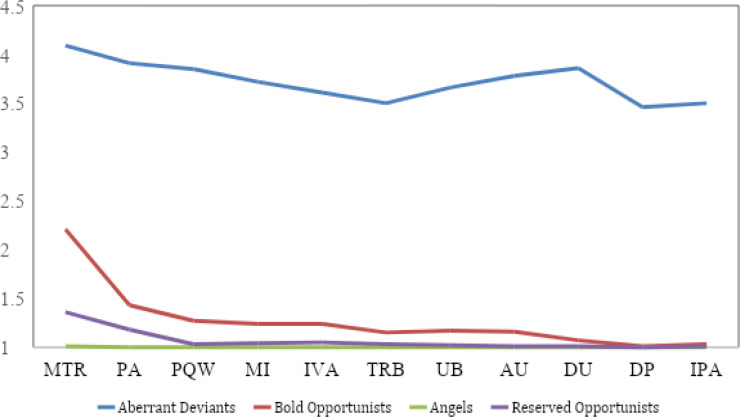
Means plot for each latent profile. TRB = theft and related behaviors, DP = destruction of property, MI = misuse of information, MTR = misuse of time and resources, UB = unsafe behavior, PA = poor attendance, PQW = poor quality work, AU = alcohol use, DU = drug use, IVA = inappropriate verbal behaviors, IPA = inappropriate physical actions.

The second profile constituted the largest portion of participants at 39% (*n*= 191) of the sample. The second profile more closely resembled the shape of mean levels found in the aberrant deviants’ profile than the fourth profile with the exception of drug use (see Figure 3). The second profile also differed from the angels and fourth profile in that it was the only profile other than the aberrant deviants that reported a level of any CWB above its mean (misuse of time and resources *z*= .14; Figure 2). Accordingly, the second profile was labeled, “bold opportunists.”

Lastly, the fourth profile constituted the remaining 33% (*n*= 162) of the sample. In terms of shape, this profile shared its two highest levels of CWBs with the bold opportunists (misuse of time and resources and poor attendance), however, it demonstrated minimal divergence in level with the angels’ profile. Indeed, the only meaningful deviation from the angels was higher levels of misuse of time and resources and poor attendance, and slightly higher inappropriate verbal actions. This pattern produced a shape that was ultimately distinct from the other three profiles but broadly marked by mild counterproductivity in the most commonly reported dimensions. Considering the relatively low levels of CWBs, the large proportion of participants classified in this profile, and the CWBs reported being both common and relatively mundane in form, this profile was labeled “reserved opportunists.” In sum, results from the LPA were generally consistent with the proposition that latent subpopulations exist, in terms of individuals’ “signature” patterns across different CWBs.

### Analyses of Variance

Research question 3 asked whether dark triad traits were related to profile membership. A multivariate analysis of variance (MANOVA) was conducted to test for mean differences on dark triad traits between profiles, and subsequent between-subjects effects were calculated for each dark triad trait with post-hoc comparisons between profiles. Prior to conducting the MANOVA, the Levene statistic was calculated for each dark triad trait in order to test for violations of homogeneity of variance. Findings revealed that this assumption was violated only for narcissism (*F*(3, 485) = 3.31, *p*= .02). Nevertheless, all post-hoc comparisons were tested using the Games-Howell statistic for the sake of consistency, as this statistic has shown to be robust under similar conditions (Wilcox, 1987).

**Figure 4:**
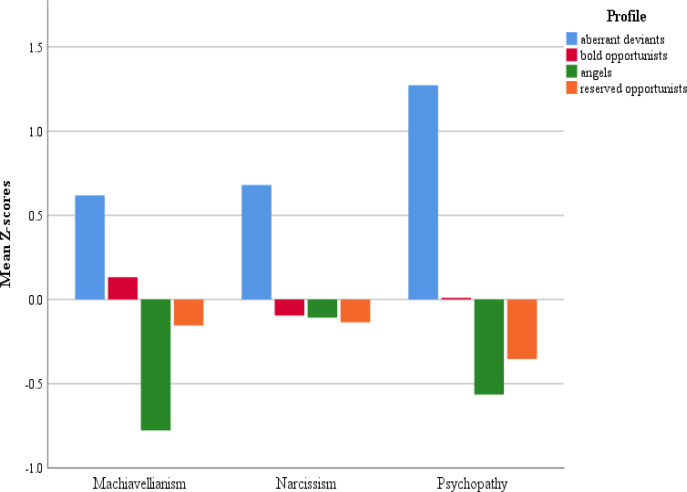
Mean profile z-scores for each dark triad trait.

Results from the omnibus MANOVA showed differences between profiles in the mean levels of the overall dark triad scale (*F* (3, 483) = 24.08, *p*< .001), and on the individual traits of Machiavellianism (*F* (3, 485) = 27.90, *p*< .001 *η^2^* = .15), narcissism (*F* (3, 485) = 12.96, *p*< .001 *η^2^* = .07), and psychopathy (*F* (3, 485) = 72.01, *p*< .001 *η^2^* = .31). As shown in Table 3, all comparisons between profiles on Machiavellianism were statistically significant (*M_aberrant deviants_* = 3.32, *M_bold opportunists_* = 2.94, *M_reserved opportunists_* = 2.72, and *M_angels_* = 2.23), and all comparisons on psychopathy were statistically significant (*Maberrant deviants* = 3.08, *Mbold opportunists* = 2.16, *M_reserved opportunists_* = 1.89, and *M_angels_* = 1.74) with the exception of angels-reserved opportunists (*p*= .36). For narcissism, the aberrant deviants’ profile (*M_aberrant deviants_* = 3.10) was significantly different from all other profiles (*M_bold opportunists_* = 2.60, *M_reserved_ opportunists* = 2.57, and *M_angels_* = 2.59); however, all remaining comparisons revealed nonsignificant differences. Figure 4 shows the profiles’ mean *z*-scores for each dark triad trait.

**Table 3:** Profile Means and Standard Deviations for Dark Triad Traits and Previous History

Profile	Mach	Narc	Psyc	Disciplined	Fired	Arrested
Aberrant Deviants	3.32(.74)	3.10(.52)	3.08(.51)	1.41(1.2)	1.19(1.14)	1.07(1.16)
Bold Opportunists	2.94(.72)	2.60(.66)^a^	2.16(.63)	.50(.92)	.46(.77)^a^	.24(.65)^a^
Angels	2.23(.84)	2.59(.67)^a^	1.74(.65)^a^	.04(.21)	.13(.39)^b^	.01(.12)^b^
Reserved Opportunists	2.72(.70)	2.57(.65)^a^	1.89(.61)^a^	.19(.51)	.29(.61)^a,b^	.14(.48)^a,b^
Total	2.83(.79)	2.66(.66)	2.14(.73)	.46(.89)	.46(.81)	.29(.73)

*Note*. Values in parentheses are standard deviations. Values with the same superscript refer to values that were not significantly different from each other. All other values were significantly different (p < .05). Mach = Machiavellianism, Narc = narcissism, Psych = psychopathy, Disc = previous disciplinary experiences, Fired = previous number of terminations, and Arrest = previous arrest history.

Research question 4 asked whether there were differences between profiles in terms of previous disciplinary action by employer. To examine this question, separate analyses of variance (ANOVAs) were conducted for the two disciplinary action items. Again, the Games-Howell statistic was used for all pairwise comparisons as there were unequal variances and sample sizes between profiles. For the first item, referring to previously being disciplined by an employer, the omnibus test for differences across profiles was statistically significant (*F*(3, 485) = 46.27, *p*< .001 *η^2^* = .22) and all pairwise comparisons were statistically significant. Aberrant deviants (*M*= 1.41) reported the most instances of discipline while the angels (*M*= .04) reported the fewest. For the second item, referring to previously being fired/terminated by an employer, results also revealed statistically significant differences across profiles (*F* (3, 485) = 29.06, *p*< .001 *η^2^* = .15). The aberrant deviants (*M*= 1.19) were significantly different from all other profiles and the bold opportunists (*M*= .46) and angels (*M*= .13) were significantly different, all other comparisons were nonsignificant (see Table 3).

**Figure 5:**
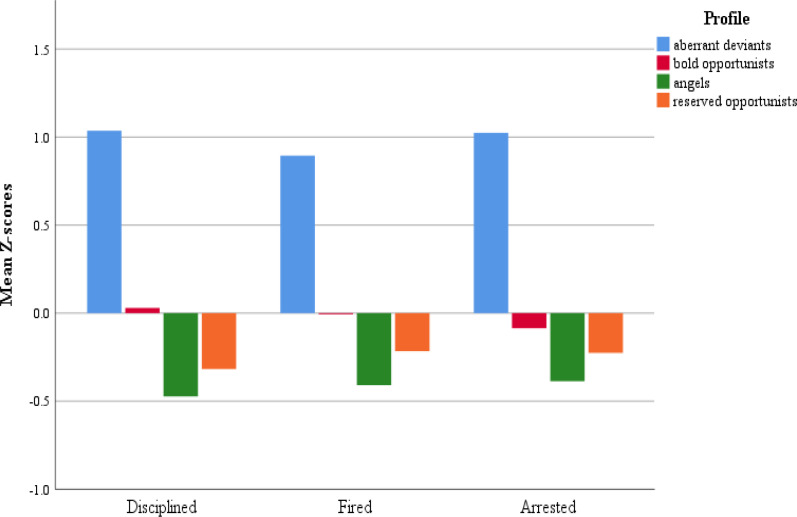
Mean profile z-scores for previous disciplinary and arrest histories.

Research question 5 asked whether profiles differed on previous arrest history. ANOVA’s omnibus test showed that there were statistically significant differences across profiles (*F* (3, 485) = 39.83, *p*< .001 *η^2^* = .20). Post-hoc comparisons revealed results identical to the pattern observed in the previously fired/terminated item, whereby the aberrant deviants (*M*= 1.07) were significantly different from all other profiles and the bold opportunists (*M*= .24) and angels (*M*= .01) were significantly different. All other comparisons were nonsignificant (see Table 3). Figure 5 displays each profile’s mean levels on the three sanction items.

## Discussion

The aim of the current research was to investigate whether subpopulations of counterproductive employees can be identified using a person-oriented approach. Results indicated that the co-occurrence of CWBs varies across individuals, allowing individuals to be grouped into distinct profiles of counterproductive behavior. Specifically, an LPA converged on a four-profile solution, and subsequent analyses revealed that profiles differed on three personality variables, and on likelihood of disciplinary action and arrest. These results suggest that employees differ, not only in the “amount” of their CWB, but also in its kind. That is, employees can be meaningfully categorized on the basis of their signature patterns across specific CWBs. Further, the different types of employees are differentially frequent within the broader population.

The profile characterized by the highest levels of CWBs (“aberrant deviants”), constituting 14% of the sample, also showed the highest mean scores for all dark triad traits. Members of this profile also reported experiences of being disciplined or terminated by employers, and having been arrested, at more than double rate of any other profile (see Table 3). The elevated means of the aberrant deviants’ profile may initially appear to reflect individuals that are just more counterproductive than individuals in the other profiles, but closer inspection reveals that the shape of the profile differs from the others in interesting ways.

First, the aberrant deviants’ third most frequent counterproductive behavior, drug use, was one of the least common for all other profiles. Angels and reserved opportunists reported almost never engaging in drug use while the bold opportunists reported drug use as the third least common CWB. Second, while the within-profile variance for each CWB dimension was greater for the aberrant deviants than the other profiles (indicating greater heterogeneity within profile), the aberrant deviants’ means across CWBs differed less between the most and least commonly engaged CWBs compared to the bold opportunists but more than the angels and reserved opportunists. Thus, the aberrant deviants and bold opportunists were slightly more selective in the types of CWB they engaged in compared to the other two profiles. Third, the frequencies of the most serious CWBs were at strikingly high levels in the aberrant deviants’ profile (although the bold opportunists also reported some frequency of theft and inappropriate verbal actions). All CWBs are, by definition, detrimental to the well-being of the organization and its stakeholders, but distinctions among more and less serious forms have been noted (Robinson & Bennett, 1995). The aberrant deviants’ profile was characterized by a high frequency of more mundane CWBs (e.g., poor attendance), but also less common and more serious CWBs (e.g., inappropriate physical actions and drug use). Members of this subgroup appear to not only be more counterproductive in a general sense, but also more extreme in their choices of CWB.

Aberrant deviants had the highest mean scores on all three dark triad traits, which was consistent with previous research that examined these relations from a variable-oriented perspective (O’Boyle et al., 2012). Additionally, aberrant deviants reported significantly more experiences of being disciplined and fired by employers, as well as more arrests. This study’s inclusion of nonwork deviance is not common in organizational research but produced results in line with other research suggesting a role of dark triad traits in social deviance more broadly (Wu & LeBreton, 2011). For example, individuals high in Machiavellianism are often characterized as manipulative, cynical, and concerned with their reputation, and those high in psychopathy are characterized by a lack of empathy and a callous orientation towards others. Given that people carry their personalities with them between work and personal life, it seems probable that those scoring high in Machiavellianism and psychopathy display these tendencies at work, at social events, at school, and in most other settings. Associations between Machiavellianism, psychopathy, and narcissism, and maladaptive social behavior are well documented. A recent meta-analysis by Muris et al. (2017) reported positive correlations between the dark triad and a variety of negative psychosocial outcomes, such as aggression/delinquency and antisocial tactics.

The bold opportunists’ profile described the largest proportion of participants (37%) and was marked by a pattern of CWB frequencies that appears to be less serious than that of the aberrant deviants but still higher than the other two profiles in terms of common but less serious CWBs. Nevertheless, bold opportunists can be differentiated from angels and reserved opportunists in more than just frequency of common CWBs, as more than 50% of bold opportunists reported engaging in inappropriate verbal actions, theft and related behaviors, and misuse of information. Thus, members of this group deviated from the otherwise similar pattern of the reserved opportunists in their proclivity to engage in more serious CWBs. This distinction between the bold opportunists and reserved opportunists is reinforced by the finding of statistically significant differences in their mean Machiavellianism and psychopathy scores, where bold opportunists had higher scores than either the reserved opportunists or the angels. Finally, bold opportunists reported more experiences of being disciplined than the angels or reserved opportunists, as well as more experiences of being fired or arrested than the angels.

The reserved opportunists were the second most frequent profile (33%) and were characterized by CWB frequencies below the sample means (see Figure 2). As the label implies, members of this profile reported few CWBs, and those that were reported were among the more common and mundane types – misuse of time and resources and poor attendance (Gruys & Sackett, 2003). Reserved opportunists had significantly higher scores on Machiavellianism and reported receiving more organizational discipline than angels but shared similar mean levels of all other non-CWB variables (e.g., narcissism and previous arrests) with the angels. The reserved opportunist profile appears to describe individuals without elevated dark triad scores, and less disciplinary experience, who engage in CWBs only selectively, perhaps as permitted or induced by infrequent situational conditions.

The angels’ profile was characterized by near-zero CWB frequencies, and was also the smallest group at 14% of the sample. Correspondingly, this profile had the lowest means for previous sanction and arrest histories and was significantly lower in Machiavellianism than all other profiles, and significantly lower than the bold opportunists and aberrant deviants in psychopathy. Its members scored below average on dark triad traits, described themselves on the CWB measure as exceptionally rule-abiding, and had low rates of employer sanction and arrest. Of course it is possible that angels simply engage in more impression management than members of other profiles, and under-report their counterproductive behavior, but previous research suggests that is unlikely to explain the current results. For instance, scientists using the counterproductive behavior checklist (CWB-C) found that many CWBs are very rarely endorsed, and that even the most common and mild CWB in their measure, taking a longer break than permitted, was not universally reported (e.g., 62% of their sample; Spector et al., 2006). Additionally, a study using the same measure as the current study (Gruys & Sackett, 2003) found composite subscale means very similar to the ones reported in the current study, which suggests that current participants are unlikely to have impression-managed any more than participants in previous research. Coupled with the careless responding screen performed prior to data analysis, it is suggested here that the angels’ profile largely reflects rule-bound employees.

Dark triad traits were positively related to CWBs across the full sample. This finding is consistent with prior variable-centered research evidencing positive correlations between dark triad traits and CWBs (O’Boyle et al., 2012), as well as a litany of more broadly negative behaviors and outcomes (see Furnham et al., 2013 for a review). Taken as a whole, our results suggest that considerations of kind should accompany the substantial body of knowledge regarding degree.

### Practical Implications

The most pertinent implication of the current study’s findings for practitioners is that not all counterproductive employees are the same. Specifically, subgroups of employees perform different amounts and types of CWBs. When implementing a selection test for the purpose of reducing CWBs in the workplace (e.g., an integrity test), the goal is to identify and screen out individuals that engage in CWBs. In the current study’s data, this may be the individuals categorized into the aberrant deviants’ profile, which reported high frequencies of CWBs on average across all types of CWBs. Many of these instruments contain items regarding petty behaviors from past jobs or contain items that target specific types of CWB like theft or absenteeism (see Van Iddekinge et al., 2012). One implication from the current study is that individuals that may report CWBs of the most common types (e.g., bold and reserved opportunists) could be screened out although they fall below the mean on these dimensions of CWB, and report very little frequency in regards to engaging in more serious CWBs (e.g., inappropriate physical actions or drug use). For example, the reserved opportunists reported engaging in misuse of time and resources and poor attendance; however, their means on these two dimensions were more than half a standard deviation below the total mean. The consequences of such screening procedures in this scenario could include a failure to hire individuals that are rarely counterproductive and very rarely commit serious CWBs, and potentially penalizing individuals for reporting common work behaviors honestly. Additionally, our results with dark triad measures suggests that personality-based selection methods may be differentially predictive of specific types of counterproductive employees. Additional research is needed to more precisely link configurations of personality traits with configurations of CWBs.

Another implication from the current study’s findings pertains to the development and implementation of post-selection CWB interventions. Although the systematic study of such interventions on incumbents is less frequently reported than interventions during the selection phase with applicants, organizations sometimes incorporate deterrents to CWB whether during the onboarding process or within company policies and procedures. For example, organizations may use random drug testing or auditing a cash register at the end of the shift in order to evaluate whether CWB has occurred. When particular issues are in need of immediate redress, especially when perpetrators may not be individually identifiable, managers may incorporate interventions for targeting the behavior. Organizations have turned to a number of surveillance or monitoring programs that aim to deter CWBs as mundane as making an unauthorized stop at Starbucks while on a delivery route or checking a personal e-mail account while at work – CWBs that the current study and previous ones (e.g., Gruys & Sackett, 2003; Spector et al., 2006) have found to be the most common. These electronic performance monitoring (EPM) programs have shown promise at enhancing employee performance and reducing CWB, however, accumulating evidence has found they can detract from individual and group-level outcomes, such as commitment, satisfaction, fairness perceptions, and may even increase the likelihood of employees engaging in CWBs (Tomczak et al., 2018). There is likely little debate as to whether serious CWBs, such as enacting violence or stealing, should require interventions and consequences, and it is reasonable that engaging in performance detracting, off-task behaviors is virtually always deleterious to task performance.

The current research has shown, however, that while some groups of employees (e.g., aberrant deviants) engage in extreme CWBs as well as more frivolous CWBs, other groups (e.g., bold and reserved opportunists) perform the most frequent and less serious CWBs almost exclusively. Thus, interventions targeting those frequent and less serious CWBs via monitoring technologies will inevitably capture individuals from all groups. If the goal in instituting an internet monitoring system is to reduce cyberloafing, then flagging and disciplining individuals may accomplish that goal. Alternatively, if managers aim to reduce counterproductivity more broadly and assume that the target of their monitoring is just a sample of such counterproductivity, the current research suggests that such an assumption may be misguided, or at least inefficient. Additionally, future research is needed to investigate the possibility that different CWB types may be differentially deterrable, or otherwise differentially responsive to situational interventions. In conclusion, organizations should develop interventions that account for the distinction among types of counterproductive employees so that employees that most expose the organization to legal, lifethreatening, and/or serious financial problems (aberrant deviants in the current study) are targeted without expense to the less counterproductive employees.

### Limitations and Future Research

A number of limitations of the current study bear mentioning. First, the use of a heterogeneous sample that pulled from many different occupations and industries may enhance generalizability, but it also limited the study’s internal control. Person-oriented approaches to CWB would benefit from future research that investigates the possibility of subgroups in workplaces that provide some level of commonality. For instance, similar occupations, particularly within the same organization, may have fewer discrepancies in the number and types of situational constraints that facilitate or limit counterproductive behaviors. Future investigations may tease apart whether these subgroups differ according to the type of work or employing organization.

This study employed Mechanical Turk to source employees and used multiple methods of screening to eliminate careless responders. Nevertheless, all data were self-reported and regarded socially undesirable traits, behaviors, and history. This may have (a) inflated relationships among variables by using a single source, and/or (b) restricted variance and lowered mean levels (i.e., downwardly biased estimates) of the socially undesirable variables. Thus, our design may have limited our ability to distinguish between counterproductive profiles, which we believe may make our finding of four profiles all the more notable. Additionally, the LPA method that we employed is only one of several available analyses (e.g., hierarchical agglomerative and K-means cluster analysis, multidimensional scaling, Q factor analysis, etc.) and a different analytic method might have produced a different profile solution. Future research using multiple methods of measurement/sources and differing person-oriented analytic strategies would be informative as to the possibility of even more potential subgroups of counterproductive employees.

Another important area for future research would be the investigation of profiles using alternative CWB measures. As previously discussed, there are at least three commonly used instruments to measure CWBs, each with its own uniqueness but with overlap across measures. The Gruys and Sackett (2003) measure used in this study was chosen on the basis of its comprehensiveness, but the 11-factor structure proposed by its authors demonstrated only marginal fit to the current data. Findings from its use should be compared to those from other measures, such as the Bennett and Robinson (2000) and Spector et al. (2007) instruments, as shown in Marcus et al. (2016). Finding evidence of generalizability across measures would contribute to the validity of the four-profile taxonomy identified in the current study.

## Conclusion

There is a long and storied history of studying individual differences in psychology. This tradition, in both the conceptualization of the individual and the linking of individual differences to external criteria, is most heavily represented by the variable-centered approach. Nevertheless, one of the earliest pioneers of individual differences’ research in personality psychology, Gordon Allport (1930), noted the distinction between within-person and between-persons differences:

Now if we visualize several distribution curves for several traits, and plot on the base line of each the position attained by Alice, we find that the significant thing for our understanding of Alice is not her position in each curve or the average of her positions in all the curves, but rather the profile which would result from connecting her positions in the different curves. This qualitative pattern is more significant than measurements on anyone or on all of the isolated traits. In short, the natural point of reference in understanding Alice is Alice herself, and not the population at large. (Allport, 1930, p. 124)

Framing variables as the centerpiece of psychological investigations has proven undeniably productive in the study of human behavior, as it forms the background to almost all current research into personality – behavior relationships. There are, however, increasingly vocal calls to supplement this variable-centered research with person-oriented research that can offer different, complementary perspectives (Wang & Hanges, 2011). Although these approaches are now being applied to the study of personality and work behavior (e.g., Nguyen et al., 2021), we could not find any personoriented approach used to identify subpopulations of counterproductive employees.

The present study found four profiles of counterproductive employees in terms of their patterns of CWB, and further substantiated by patterns of theoretically supported relationships with dark triad traits and previous work and non-work experiences. That is, these profiles were empirically derived from latent profile analysis of self-reported CWBs, and then further supported by relationships with other criteria – all of which supported and extended extant variable-centered research. The findings of this study do not appear to conflict with the current understanding of personalityCWB relationships, however, there are contributions and possible future avenues of research that bear mention.

Counterproductive work behaviors differ in both amount and kind. This study is among the first to demonstrate that counterproductive employees also differ in kind and not just their frequency in engaging in CWBs. Specifically, this study found that subgroups of employees can be separated on the basis of their frequency, or level, of CWBs, and the kinds, or shape, of CWBs they report. This finding is important because it suggests that not all counterproductive employees are the same, whether in terms of their personality or previous history, or in terms of the way in which they are counterproductive. Most importantly, the profiles identified in this study were not reflective of varying amounts of CWB (i.e., “low,” “medium,” “high”), but reflective of differing patterns and amounts of CWB. Our identification of latent subpopulations within a diverse sample of working adults may indicate a need to recalibrate scientific thinking on the nature of CWB away from a variable orientation and toward a more Gestaltist perspective.

Most research on CWB has taken a variable-centered approach, providing fruitful advancements to both theory and practice. The current study complements this growing body of research by providing the first step toward a taxonomy of counterproductive employees and linking the identified types to dark triad traits and previous disciplinary and arrest histories. As results collectively indicated the existence of four distinct profiles of counterproductivity, there are several implications for the study of CWB, as well as practical considerations for managers and consultants in terms of selection and organizational interventions.

## Data Availability

Data are available upon reasonable request from the first author.

## Action editor

Lars-Gunnar Lundh served as action editor for this article.

## Open access

This article is distributed under the terms of the Creative Commons Attribution 4.0 International License (http://creativecommons.org/licenses/by/4.0/), which permits unrestricted use, distribution, and reproduction in any medium, provided you give appropriate credit to the original author(s) and the source, provide a link to the Creative Commons license, and indicate if changes were made.
